# Genomic Surveillance for SARS-CoV-2 Variants Circulating in the United States, December 2020–May 2021

**DOI:** 10.15585/mmwr.mm7023a3

**Published:** 2021-06-11

**Authors:** Prabasaj Paul, Anne Marie France, Yutaka Aoki, Dhwani Batra, Matthew Biggerstaff, Vivien Dugan, Summer Galloway, Aron J. Hall, Michael A. Johansson, Rebecca J. Kondor, Alison Laufer Halpin, Brian Lee, Justin S. Lee, Brandi Limbago, Adam MacNeil, Duncan MacCannell, Clinton R. Paden, Krista Queen, Heather E. Reese, Adam C. Retchless, Rachel B. Slayton, Molly Steele, Suxiang Tong, Maroya S. Walters, David E. Wentworth, Benjamin J. Silk

**Affiliations:** ^1^CDC COVID-19 Response Team; ^2^Office of Advanced Molecular Detection, National Center for Emerging and Zoonotic Infectious Diseases, CDC.

SARS-CoV-2, the virus that causes COVID-19, is constantly mutating, leading to new variants (*1*). Variants have the potential to affect transmission, disease severity, diagnostics, therapeutics, and natural and vaccine-induced immunity. In November 2020, CDC established national surveillance for SARS-CoV-2 variants using genomic sequencing. As of May 6, 2021, sequences from 177,044 SARS-CoV-2–positive specimens collected during December 20, 2020–May 6, 2021, from 55 U.S. jurisdictions had been generated by or reported to CDC. These included 3,275 sequences for the 2-week period ending January 2, 2021, compared with 25,000 sequences for the 2-week period ending April 24, 2021 (0.1% and 3.1% of reported positive SARS-CoV-2 tests, respectively). Because sequences might be generated by multiple laboratories and sequence availability varies both geographically and over time, CDC developed statistical weighting and variance estimation methods to generate population-based estimates of the proportions of identified variants among SARS-CoV-2 infections circulating nationwide and in each of the 10 U.S. Department of Health and Human Services (HHS) geographic regions.* During the 2-week period ending April 24, 2021, the B.1.1.7 and P.1 variants represented an estimated 66.0% and 5.0% of U.S. SARS-CoV-2 infections, respectively, demonstrating the rise to predominance of the B.1.1.7 variant of concern^†^ (VOC) and emergence of the P.1 VOC in the United States. Using SARS-CoV-2 genomic surveillance methods to analyze surveillance data produces timely population-based estimates of the proportions of variants circulating nationally and regionally. Surveillance findings demonstrate the potential for new variants to emerge and become predominant, and the importance of robust genomic surveillance. Along with efforts to characterize the clinical and public health impact of SARS-CoV-2 variants, surveillance can help guide interventions to control the COVID-19 pandemic in the United States.

With high levels of SARS-CoV-2 transmission globally, continued emergence of new variants is expected. Variants have potential impacts on COVID-19 severity, transmission, diagnostics, therapeutics, and natural and vaccine-induced immunity (*1*). The emergence and rapid expansion of multiple SARS-CoV-2 variants of interest^§^ (VOIs) and VOCs, and the potential for variants of high consequence,^¶^ (VOHCs) (Supplementary Table 1, https://stacks.cdc.gov/view/cdc/106690) indicate the need for robust genomic surveillance to monitor circulating viruses and help guide the public health response to the COVID-19 pandemic.

CDC's national SARS-CoV-2 genomic surveillance program includes genomic sequences from the National SARS-CoV-2 Strain Surveillance (NS3) program and contracted commercial laboratories. Each week, public health laboratories from all U.S. jurisdictions (50 states, the District of Columbia, and eight U.S. territories and freely associated states) are requested to submit a target number of specimens representative of the geographic and demographic diversity in each jurisdiction collected during the preceding 7 days, which can be achieved through random selection.** Specimens are submitted to CDC for assessment, sequencing, and genomic analysis. SARS-CoV-2 lineages are assigned using the Phylogenetic Assignment of Named Global Outbreak Lineages software (PANGOLIN; version 3.03; Rambaut Laboratory) (*2*).

In December 2020, CDC expanded the volume of SARS-CoV-2 sequencing through contracts with large commercial diagnostic laboratories, which were selected based on geographic coverage and specimen volume. Commercial laboratories submit random samples of geographically diverse sequences with limited demographic data to CDC weekly. Specimen sources for these laboratories include retail pharmacies, community testing sites, and inpatient and outpatient health care settings served by large commercial laboratories; any type of specimen tested for SARS-CoV-2 by reverse transcription–polymerase chain reaction (RT-PCR) may be submitted. Commercial laboratories use a variety of platforms and approaches to conduct sequencing; all SARS-CoV-2 sequence data are submitted to CDC for quality assessment, genomic analysis, and database upload. Sequences generated by both NS3 and commercial laboratories are deposited into public repositories (National Center for Biotechnology Information [NCBI] and Global Initiative on Sharing All Influenza Data [GISAID]). Data from genomic surveillance based on specimens received from NS3 and commercial laboratories were analyzed weekly to monitor SARS-CoV-2 variants circulating in the United States. This activity was reviewed by CDC and was conducted consistent with applicable federal law and CDC policy.^††^

The estimated proportions of variant lineages among circulating SARS-CoV-2 viruses are calculated based on specimen collection date. Proportions of all lineages accounting for >1% of sequences nationally during the preceding 12 weeks as well as all VOIs and VOCs identified among circulating viruses are estimated nationally and for all 10 HHS regions and are updated weekly to CDC’s COVID Data Tracker.^§§^

Because the proportion of sequenced SARS-CoV-2 infections varies geographically and over time, proportions of variants at the jurisdiction level and by week of specimen collection are weighted to generate population-based national and regional estimates of the proportion of each circulating variant among all SARS-CoV-2 infections. Weighting accounts for the inverse probability that 1) a specimen from a positive RT-PCR test was sequenced (w_p_), and 2) a person with SARS-CoV-2 infection was tested by RT-PCR (w_i_) (i.e., the infection was diagnosed). To calculate w_p,_ first the number of positive RT-PCR tests is divided by the number of sequences in the sample to obtain a weight to represent all RT-PCR positive cases; this weight is then adjusted for a known sampling bias (oversampling of S-gene target failure [SGTF] results by one laboratory) using a logistic regression model that assumes no sampling bias in the remainder of the laboratories. Second, w_i_ is calculated to account for variations in probability of RT-PCR testing among persons with SARS-CoV-2 infection. SARS-CoV-2 infection incidence is estimated as the geometric mean of the incidence of test-positive cases and the percentage of positive test results.^¶¶^ The estimated number of infections, divided by the number of RT-PCR–positive cases, yields w_i_. The final weight is the inverse of the probability that a person with SARS-CoV-2 infection contributes to the sample of sequences and is calculated as w_p_ multiplied by w_i_. Variance is estimated for 95% confidence intervals (CIs) using a survey design-based approach. HHS regions are designated as survey strata, and data sources within each state are designated clusters (i.e., NS3 or each commercial laboratory).

Because the time from specimen collection to sequence availability currently is approximately 3 weeks, projections extending beyond the time frame of available data are made to enable estimation of current variant proportions during this 3-week interval. These projections, termed “nowcasts,” and their 95% prediction intervals, are generated by using a multinomial logistic regression model fit to weighted sequencing data. The nowcast model is a multivariant extension to a two-variant framework previously described (*3*). Nowcast estimates are projections and might differ from weighted estimates that are subsequently generated for the same periods.***

As of May 6, 2021, a total of 177,044 SARS-CoV-2 viral sequences for specimens collected during December 20, 2020–May 6, 2021 from 55 U.S. states and territories had been generated by NS3 or reported to CDC by contract laboratories; these included 3,275 sequences from specimens collected during the 2-week period ending January 2, 2021, compared with a sixfold increase to 25,000 sequences from specimens collected during the 2-week period ending April 24, 2021 (accounting for 0.1% and 3.1% of positive RT-PCR tests reported to CDC, respectively) (Figure). The proportion of specimens with sequences varied across states (Supplementary Table 2, https://stacks.cdc.gov/view/cdc/106690); weighting methods generated regional- and national-level estimates of variant proportions over time.

**FIGURE:**
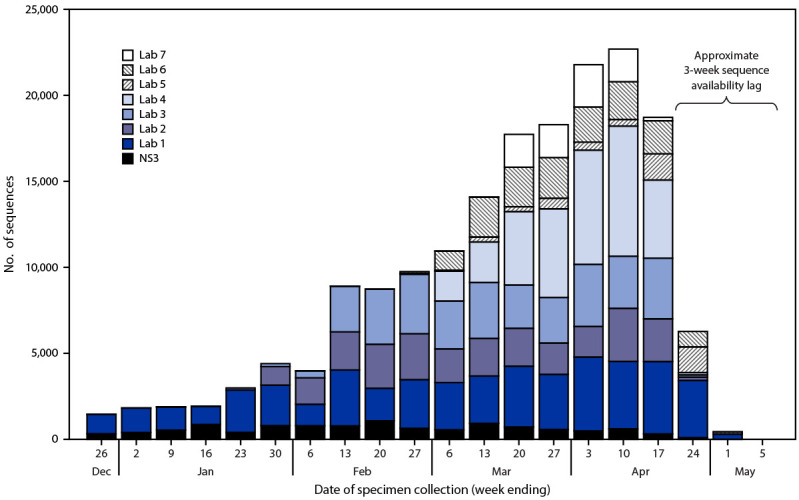
Number of SARS-CoV-2 genomic sequences generated by National SARS-CoV-2 Strain Surveillance or reported to CDC by commercial laboratories* for specimens collected December 20, 2020—May 6, 2021, by laboratory source — United States, May 6, 2021 **Abbreviation:** NS3 = National SARS-CoV-2 Strain Surveillance * Sequences generated by or reported to CDC through NS3 and contract laboratories do not include the >5,000 sequences per week produced by public health laboratories and other U.S. institutions, which are not currently integrated into CDC’s surveillance for SARS-CoV-2 variants using genomic sequencing. https://covid.cdc.gov/covid-data-tracker/#published-covid-sequences

The B.1.1.7 VOC represented an estimated 0.2% of U.S. infections during the 2-week period ending January 2 and increased to 66.0% during the 2-week period ending April 24 (Table). During this period, estimated proportions of B.1.1.7 infections varied across HHS regions, from 50.9% in HHS Region 1 to 74.1% in HHS Region 6 (Supplementary Table 2, https://stacks.cdc.gov/view/cdc/106690). This rapid expansion is consistent with a model-based prediction that B.1.1.7 could become a predominant variant (*3*). The nowcast model estimated that B.1.1.7 represents 72.4% (95% prediction interval = 67.4%–77.1%) of infections for the 2-week period April 25–May 8, 2021 (Table). The P.1 VOC first appeared the 2 weeks ending January 30; by the 2-week period ending April 24, the P.1 variant represented an estimated 5.0% of infections, ranging from 1.6% in HHS Region 3 to 7.7% in HHS Region 5 (Table). Uncertainty around point estimates, as captured by confidence and prediction intervals, differed substantially by variant, time period, and region (Table).

**TABLE:** Weighted proportions of SARS-CoV-2 infections attributable to B.1.1.7 and P.1 variants among all estimated SARS-CoV-2 infections in the United States during December 20, 2020–April 24, 2021 and nowcast* projected proportions during April 25–May 8, 2021, by 2-week period and U.S. Health and Human Services Region^†^ — United States, December 20, 2020–May 8, 2021

Variant/HHS region no.	Weighted % (95% CI)									Projected % (95% PI^§^)
	Dec 20–Jan 2	Jan 3–16	Jan 17–30	Jan 31–Feb 13	Feb 14–27	Feb 28–Mar 13	Mar 14–27	Mar 28–Apr 10	Apr 11–24	Apr 25–May 8 (nowcast^§^)
**B.1.1.7**										
**All**	**0.2 (0.1–0.4)**	**0.3 (0.1–0.9)**	**1.2 (0.7–2.1)**	**4.5 (2. 9–6.9)**	**11.4 (8.2–15.6)**	**27.3 (22.1–33.2)**	**44.6 (39.3–50.1)**	**59.5 (54.9–64.0)**	**66.0 (62.0–69.8)**	**72.4 (67.4–77.1)**
1	—^¶^	—	0.4 (0.1–2.9)	3.4 (2.0–5.7)	11.5 (7.6–17.2)	21.6 (16.1–28.4)	37.0 (30.8–43.7)	44.3 (35.4–53.6)	50.9 (43.4–58.3)	57.2 (42.6–70.2)
2	0.8 (0.3–2.2)	—	2.9 (2.3–3.7)	9.9 (1.6–42.8)	14.6 (9.0–23.0)	28.5 (23.1–34.5)	37.1 (27.4–48.0)	48.2 (37.3–59.3)	54.5 (36.6–71.4)	52.8 (31.8–72.7)
3	—	0.7 (0.1–8.2)	0.9 (0.3–2.7)	2.1 (0.7–5.9)	9.8 (4.8–19.0)	25.0 (18.8–32.5)	46.2 (39.1–53.5)	62.1 (55.8–68.0)	66.5 (61.0–71.6)	73.8 (60.5–86.8)
4	—	0.6 (0.1–3.9)	1.8 (0.4–8.3)	6.6 (2.7–15.1)	15.0 (6.8–29.8)	36.5 (22.7–52.9)	54.5 (43.2–65.3)	65.8 (59.9–71.2)	70.5 (64.8–75.5)	78.6 (67.3–89.1)
5	0.2 (0.0–3.6)	0.5 (0.1–4.7)	0.6 (0.3–1.6)	3.1 (1.7–5.5)	8.1 (2.9–21.1)	26.5 (15.0–42.4)	49.9 (32.9–67.0)	67.6 (54.8–78.3)	73.1 (59.7–83.3)	79.1 (67.4–90.7)
6	—	0.1 (0.0–2.6)	1.4 (0.9–2.0)	4.2 (2.7–6.6)	14.3 (7.4–26.0)	33.0 (24.9–42.3)	50.5 (40.5–60.5)	69.3 (65.6–72.7)	74.1 (70.6–77.2)	82.5 (68.6–94.3)
7	—	—	—	1.6 (0.3–8.1)	7.7 (2.6–20.3)	13.6 (2.9–45.6)	13.6 (2.9–45.6)	63.8 (53.3–73.2)	72.4 (59.7–82.3)	77.0 (61.5–92.3)
8	1.7 (0.1–32.5)	—	—	2.9 (0.5–14.0)	4.7 (1.8–12.1)	18.7 (12.0–28.1)	35.4 (24.1–48.5)	53.7 (43.2–63.8)	56.8 (45.9–67.0)	63.6 (48.7–79.5)
9	—	—	0.3 (0.3–0.4)	1.8 (1.5–2.2)	4.8 (3.5–6.4)	13.1 (8.9–18.8)	25.1 (20.7–30.1)	43.4 (34.6–52.6)	57.7 (48.9–66.0)	62.6 (43.3–80.0)
10	—	—	1.9 (1.0–3.3)	0.6 (0.1–6.5)	7.9 (1.4–34.3)	10.4 (2.3–36.4)	25.9 (15.9–39.2)	36.6 (23.8–51.7)	52.3 (38.5–65.9)	65.4 (46.4–82.1)
**P.1**										
**All**	**—**	**—**	**0.1 (0.0–0.2)**	**0.03 (0.0–0.1)**	**0.1 (0.0–0.2)**	**0.6 (0.3–1.2)**	**1.6 (1.0–2.7)**	**3.6 (2.3–5.5)**	**5.0 (3.3–7.5)**	**6.2 (3.7–9.1)**
1	—	—	—	—	0.1 (0.0–0.4)	1.0 (0.2–4.6)	4.0 (1.2–12.3)	5.9 (2.4–13.4)	6.5 (2.0–19.0)	10.9 (2.1–21.3)
2	—	—	—	—	—	0.2 (0.01–5.3)	0.7 (0.3–1.7)	2.3 (1.9–2.9)	3.1 (1.4–6.5)	3.1 (0.0–13.6)
3	—	—	—	—	—	—	0.5 (0.2–0.9)	1.7 (1.2–2.3)	1.6 (0.7–3.3)	2.0 (0.0–7.9)
4	—	—	0.2 (0.0–1.0)	0.1 (0.0–0.4)	0.2 (0.2–3.0)	0.8 (0.2–3.0)	2.3 (0.8–6.2)	4.3 (2.0–9.1)	4.9 (2.5–9.6)	7.1 (1.8–14.5)
5	—	—	—	0.1 (0.0–0.7)	0.1 (0.0–1.6)	1.2 (0.1–10.2)	2.4 (0.5–11.0)	4.9 (0.9–21.9)	7.7 (1.6–30.8)	8.6 (2.3–18.6)
6	—	—	—	—	0.04 (0.0–0.1)	0.2 (0.1–0.6)	0.7 (0.4–1.3)	3.2 (1.9–5.6)	4.6 (3.3–6.2)	5.7 (0.0–14.3)
7	—	—	—	—	—	—	0.6 (0.1–3.6)	2.8 (1.1–7.0)	7.0 (1.3–29.4)	5.7 (0.0–15.4)
8	—	—	—	—	—	0.7 (0.2–2.1)	0.9 (0.6–1.3)	1.4 (0.7–2.7)	3.8 (1.9–7.5)	3.3 (0.0–10.3)
9	—	—	—	—	0.1 (0.0–0.3)	0.7 (0.3–1.4)	2.5 (1.8–3.4)	4.9 (4.1–5.9)	6.9 (3.6–12.9)	9.4 (0.0–20.0)
10	—	—	1.0 (0.1–9.1)	0.2 (0.0–9.9)	0.2 (0.0–6.6)	0.4 (0.0–3.8)	2.0 (0.5–8.4)	3.1 (1.1–8.4)	5.0 (2.7–8.9)	6.5 (0.0–17.9)

**Abbreviations:** CI = confidence interval; HHS = U.S. Department of Health and Human Services; PI = prediction interval.

* Because the time from specimen collection to sequence availability is approximately 3 weeks, “nowcast” projections (those that extend beyond the time frame of available data) were made to enable estimation of current variant proportions. Nowcasts and 95% PIs are generated using a multinomial logistic regression model fit to weighted sequencing data.

^†^ *Region 1:* Connecticut, Maine, Massachusetts, New Hampshire, Rhode Island, and Vermont; *Region 2:* New Jersey, New York, Puerto Rico, and the U.S. Virgin Islands; *Region 3:* Delaware, District of Columbia, Maryland, Pennsylvania, Virginia, and West Virginia; *Region 4:* Alabama, Florida, Georgia, Kentucky, Mississippi, North Carolina, South Carolina, and Tennessee; *Region 5:* Illinois, Indiana, Michigan, Minnesota, Ohio, and Wisconsin; *Region 6:* Arkansas, Louisiana, New Mexico, Oklahoma, and Texas; *Region 7:* Iowa, Kansas, Missouri, and Nebraska; *Region 8:* Colorado, Montana, North Dakota, South Dakota, Utah, and Wyoming; *Region 9:* Arizona, California, Hawaii, Nevada, American Samoa, Northern Mariana Islands, Federated States of Micronesia, Guam, Marshall Islands, and Palau; *Region 10:* Alaska, Idaho, Oregon, and Washington.

^§^ Nowcasts and 95% PIs are generated using a multinomial logistic regression model fit to weighted sequencing data.

^¶^ Dashes indicate that the number of estimated SARS-CoV-2 infections = 0.

## Discussion

The distribution of circulating SARS-CoV-2 variants in the United States changed rapidly during December 2020–May 2021. The expansion of the B.1.1.7 VOC to become the predominant variant in all U.S. regions within a 4-month period, and the more recent emergence of the P.1 VOC in all regions, underscore the critical need for robust and timely genomic surveillance. These findings are consistent with reports of potential increased transmission of the B.1.1.7 and P.1 variants^†††^ (*4*). In addition, there is evidence of potential impact of B.1.1.7 on diagnostics (i.e., SGTF in at least one RT-PCR–based diagnostic assay) (*5*) and disease severity and potential impact of P.1 on therapeutics and immunity (*1*). Four additional VOIs or VOCs (B.1.526, B.1.526.1, B.1.429, and B.1.427) are estimated to each account for >1% of circulating infections domestically as of the 2-week period ending April 24. Currently, there is no variant listed as a VOHC.

The findings in this report are subject to at least four limitations. First, although U.S. SARS-CoV-2 genomic sequencing has rapidly expanded in volume and in geographic coverage since late 2020, assessments of the national and regional representativeness of sequence data are needed. Second, although the weighting and variance estimation methods used for this analysis adjust these data to generate population-based estimates of variant proportions and quantify uncertainty, the methods assume that, within strata and clusters, sequence reporting is random. This assumption might be inaccurate; the true representativeness of sequenced specimens within each jurisdiction is unknown. Linking sequencing with epidemiologic data, for example from national case-based surveillance, might provide a better understanding of representativeness, so that specimen selection and weighting methods can be further adjusted as needed. Analyses at state and local levels have demonstrated the utility of linking sequencing with sentinel or population-based surveillance data to characterize new SARS-CoV-2 variants (*6*,*7*). Third, sequencing data from many state and local public health laboratories that are conducting SARS-CoV-2 surveillance sequencing apart from NS3^§§§^ are not yet available for inclusion in national estimates. Efforts to integrate such state and local genomic surveillance data into national surveillance and further improve national and regional surveillance are in progress. Finally, as sequence data become more complete over time, national and regional weighted estimates might change.

To respond to emerging SARS-CoV-2 variants, CDC rapidly expanded national genomic surveillance to monitor trends in circulating SARS-CoV-2 variants nationally and regionally. Along with efforts to characterize the clinical and public health impact of variants, surveillance can help guide interventions to mitigate the COVID-19 pandemic in the United States by informing prevention strategies (e.g., enhanced vaccination coverage efforts) and clinical management decisions (e.g., monoclonal antibody distribution).

**Summary**

**What is already known about this topic?**

SARS-CoV-2 variants have the potential to affect transmission, disease severity, diagnostics, therapeutics, and natural and vaccine-induced immunity.

**What is added by this report?**

CDC’s genomic surveillance for SARS-CoV-2 variants generates population-based estimates of the proportions of variants among all SARS-CoV-2 infections in the United States. During April 11–24, 2021, the B.1.1.7 and P.1 variants represented an estimated 66.0% and 5.0% of U.S. infections, respectively, demonstrating the potential for new variants to emerge and become predominant.

**What are the implications for public health practice?**

Robust genomic surveillance can help guide prevention strategies (e.g., enhanced vaccination coverage efforts) and clinical management decisions (e.g., monoclonal antibody distribution) to control the COVID-19 pandemic in the United States.

## Footnotes

*
https://www.hhs.gov/about/agencies/iea/regional-offices/index.html

† A variant for which there is evidence of an increase in transmissibility, more severe disease (e.g., increased hospitalizations or deaths), significant reduction in neutralization by antibodies generated during previous infection or vaccination, reduced effectiveness of treatments or vaccines, or diagnostic detection failures.

§ A variant with specific genetic markers that have been associated with changes to receptor binding, reduced neutralization by antibodies generated against previous infection or vaccination, reduced efficacy of treatments, potential diagnostic impact, or predicted increase in transmissibility or disease severity.

¶ A variant with clear evidence of significantly reduced effectiveness of prevention measures or medical countermeasures relative to previously circulating variants.

**
https://www.aphl.org/programs/preparedness/Crisis-Management/Documents/2021.04.09_NS3_REVISED.pdf

†† 45 C.F.R. part 46.102(l)(2), 21 C.F.R. part 56; 42 U.S.C. Sect.241(d); 5 U.S.C.0 Sect.552a; 44 U.S.C. Sect. 3501 et seq.

§§
https://covid.cdc.gov/covid-data-tracker/#variant-proportions

¶¶
https://www.medrxiv.org/content/10.1101/2020.10.07.20208504v2.full

*** Supplementary materials, including R code and sample data, are available at https://github.com/CDCgov/SARS-CoV-2_Genomic_Surveillance.

†††
https://virological.org/t/genomic-characterisation-of-an-emergent-sars-cov-2-lineage-in-manaus-preliminary-findings/586

§§§
https://covid.cdc.gov/covid-data-tracker/#published-covid-sequences
